# Cardiac Complications of Diabetes

**DOI:** 10.1155/2018/8578394

**Published:** 2018-04-03

**Authors:** Charbel Abi Khalil, Jassim Al Suwaidi, Marwan Refaat, Kamel Mohammedi

**Affiliations:** ^1^Department of Medicine and Genetic Medicine, Weill Cornell Medicine-Qatar, Doha, Qatar; ^2^Adult Cardiology, Heart Hospital, Hamad Medical Corporation, Doha, Qatar; ^3^Department of Internal Medicine, Cardiovascular Medicine/Cardiac Electrophysiology, American University of Beirut Faculty of Medicine and Medical Center, Beirut, Lebanon; ^4^Hôpital Haut-Lévêque, Service d'Endocrinologie, Diabétologie, Nutrition, Bordeaux, France; ^5^Faculté de Médecine, Université de Bordeaux, Bordeaux, France; ^6^Centre de Recherche INSERM, Université de Bordeaux U1219 “Bordeaux Population Health”, Bordeaux, France

Type 2 diabetes (T2D) is reaching epidemic proportions through most regions of the world. In a large population health study, the global age-standardized diabetes prevalence increased from 4.3% in 1980 to 9.0% in 2014 in men, and from 5.0% to 7.9% in women [1]. Potential causes for the higher incidence of T2D worldwide are increased survival of the elderly as well as epidemic bursts of obesity and sedentary lifestyle at all age ranges [2]. Diabetes is associated with a higher incidence of cardiovascular events, which results in a higher rate of mortality [3]. In patients without a history of cardiovascular disease, the 7-year risk of myocardial infarction (MI) is as high as 20% in patients with T2D as compared to only 3.5% in people without diabetes [4]. T2D also increases the risk of ischemic stroke by 35% as demonstrated in the large international case-control INTERSTROKE study [5] and the risk of peripheral artery disease (PAD) by 2- to 4-fold [6].

However, diabetes-related cardiac complications have a distinct pathophysiological background and clinical presentation. In order to understand the deleterious effect of hyperglycemia on the cardiovascular system, we propose in this special issue to highlight on all aspects of diabetes and cardiovascular diseases' interaction: epidemiology, pathophysiology, clinical manifestations, diagnosis, and treatment in 4 reviews and 6 original articles.

In their review, D. Huang et al. revisited the basic pathophysiological and epidemiological data regarding macrovascular complications in patients with diabetes and prediabetes. In fact, hyperglycemia induces low-grade inflammation and triggers the release of reactive oxygen species, which activates several pathways involved in endothelial dysfunction. Among those pathways, the formation of advanced glycation end-products (AGEs) plays a major role in initiating cardiovascular complications of diabetes. In this issue, A. Guerin-Dubourg reported that plasma concentrations of AGEs, including fructosamine, glycated albumin, and fluorescent ischemia-modified albumin, were increased in diabetic patients with established cardiovascular complications. N. El-Najjar et al. showed that hyperglycemia also induces calcium (Ca^2+^) signaling disruption in vascular smooth cells, mainly by inhibiting the passive endoplasmic reticulum Ca^2+^ leak and the sarcoplasmic reticulum Ca^2+^ -ATPase.

Diagnosing cardiac complications of diabetes can be challenging since clinical manifestations can be sometimes silent, especially at early stages. However, considering all patients with diabetes as a coronary artery disease-risk equivalent represents a strain on the healthcare system and exposes patients to unnecessary invasive exams. A. I. Guaricci et al. concluded in their systematic review of asymptomatic coronary atherosclerosis assessment that cardiac computed tomography angiography might be an adequate preliminary noninvasive test that helps explore the atherosclerotic diffusion and establish the risk of future coronary events in patients with type 2 diabetes.

Mortality in patients with diabetes is a serious concern. However, little is known in elderly polyvascular patients with T2D. J. L. Clua-Espuny reports in this special edition that the prevalence of T2D in those complex patients is above 50%. Interestingly, diabetes per se does not increase the mortality risk in this particular population, but the presence of other comorbidities such as cognitive impairment, lack of aspirin treatment, and the presence of heart failure does. S. G. Al-Kindi et al. tested the prognostic utility of a novel, easy-to-use, biomarker: the red cell distribution width (RDW). In fact, a high RDW is associated with a higher cardiovascular mortality risk, independent to other diabetes comorbidities.

Individuals with T2D are predisposed to arrhythmias, including atrial fibrillation. In their retrospective analysis of direct current cardioversion (DCCV) data, H. Soran et al. showed that diabetes was an independent risk factor for failure of DCCV in atrial fibrillation patients. Further analysis revealed that HbA_1C_ was also a predictor for an immediate failure, on top of conventional factors such as the atrial size, the left ventricular ejection fraction, and previous DCCV failures.

Finally, 2 review papers tackled the treatment of T2D and cardiovascular outcome. M. P. Nasrallah et al. nicely summarized all trials conducted to establish either the safety or protection of antidiabetic drugs, from the old UKPDS and STOP-NIDDM to the recent SGLT2 inhibitors RCTs. D. von Lewinski et al.'s review focused on the relationship between new antihyperglycemic drugs and heart failure (HF), in the light of new studies such as EMPA-REG trial showing a decrease in HF mortality in patients using empagliflozin, which is opposed to other studies such as SAVOR-TIMI that suggested an increase in HF risk with glitazones.

It is expected that T2D will impose an enormous strain on healthcare in the future and result in mortality excess of millions around the world. The latter can only be partially reversed if education, prevention, early diagnosis, and appropriate treatment of cardiovascular complications are strictly applied.
